# Identification and Validation of lncRNA-AC087588.2 in Lung Adenocarcinoma: A Novel Prognostic and Diagnostic Indicator

**DOI:** 10.3389/fmolb.2022.923584

**Published:** 2022-06-13

**Authors:** Xiulin Jiang, Xi Chen, Jishu Guo, Fan Zhou, Jun Pu, Luciano Mutti, Xiaoqun Niu

**Affiliations:** ^1^ Key Laboratory of Animal Models and Human Disease Mechanisms of Chinese Academy of Sciences and Yunnan Province, Kunming Institute of Zoology, Kunming College of Life Science, University of Chinese Academy of Sciences, Beijing, China; ^2^ Department of Neurosurgery, The Second Affiliated Hospital of Kunming Medical University, Kunming, China; ^3^ Institute for Ecological Research and Pollution Control of Plateau Lakes, School of Ecology and Environmental Science, Yunnan University, Kunming, China; ^4^ Hematology and Rheumatology Department, The Pu’er People’s Hospital, Puer, China; ^5^ Sbarro Institute for Cancer Research and Molecular Medicine, Center for Biotechnology, College of Science and Technology, Temple University, Philadelphia, PA, United States; ^6^ Department of Respiratory Medicine, Second Hospital of Kunming Medical University, Kunming, China

**Keywords:** lncRNA, lung adenocarcinoma, prognosis biomarker, immune infiltration, ceRNA, cell proliferation, cell migration

## Abstract

Lung adenocarcinoma (LUAD) is the most common histological lung cancer, and it is the leading cause of cancer-related deaths worldwide. Long non-coding RNAs (lncRNAs) have been implicated in the initiation and progression of various cancers. LncRNA-AC087588.2 (ENSG00000274976) is a novel lncRNA that is abnormally expressed in diverse cancer types, including LUAD. However, the clinical significance, prognostic value, diagnostic value, immune role, and the potential biological function of AC087588.2 LUAD remain elusive. In this study, we found that AC087588.2 was upregulated and associated with a poor prognosis in LUAD. In addition, univariate and multivariate Cox regression analysis indicated that AC087588.2 could be an independent prognostic factor for LUAD. Functionally, the knockdown of AC087588.2 restrained LUAD cell proliferation and migration *in vitro*. Finally, we constructed a ceRNA network that included hsa-miR-30a-5p and four mRNAs (ANLN, POLR3G, EHBP1, and ERO1A) specific to AC087588.2 in LUAD. The Kaplan–Meier survival analysis showed that lower expression of hsa-miR-30a-5p and higher expression of ANLN, POLR3G, EHBP1, and ERO1A were associated with adverse clinical outcomes in patients with LUAD. This finding provided a comprehensive view of the AC087588.2-mediated ceRNA network in LUAD, thereby highlighting its potential role in the diagnosis and prognosis of LUAD.

## Introduction

Lung cancer includes small cell lung carcinoma (SCLC) and non-small cell lung carcinoma (NSCLC). NSCLC includes lung adenocarcinoma (LUAD), lung squamous cell carcinoma (LUSC), and large-cell lung carcinoma. The NSCLC cancer accounts for approximately 85% of all cases (Molina et al., 2008). Although the treatment of LUAD has improved, the new LUAD pathogenesis and noninvasive diagnostic biomarkers are still needed. Therefore, the discovery of potential key prognostic markers with more characteristics and value will help early prediction and treatment of LUAD at the molecular level.

As a newly discovered non-coding RNA, the length of lncRNA usually exceeds 200 nt without protein-coding capacity (Deng et al., 2020). Emerging evidence demonstrates that lncRNA is involved in various physiological and pathological processes, including lung cancer (Lu et al., 2017). For instance, Wu et al. found that linc00673 was highly associated with poor prognosis in NSCLC. A further study showed that, by sponging miR-150-5p and upregulating ZEB1 expression, linc00673 promotes NSCLC proliferation, migration, invasion, and epithelial-mesenchymal transition (Lu et al., 2017). LncRNA ZEB1-AS1 was found to be correlated with the EMT process and adverse prognosis in LUAD (Li et al., 2016). Shen et al. found that lncRNA FEZF1-AS1 was increased in NSCLC tissues compared with adjacent normal tissues. Mechanism research indicated that, by regulating the wnt pathway, FEZF1-AS1 inhibited the EMT of NSCLC (He et al., 2017). We recently used a new method, cross-value association analysis (CVAA), to analyze the TCGA-LUAD dataset, and identified numerous new differentially expressed genes (DEGs), including lncRNA-AC087588. However, the clinical value and biological function of AC087588.2 in LUAD have not been explored.

In the present study, we use various databases to analyze AC087588.2 expression, clinical significance, prognostic value, diagnostic value, and immune infiltration and determine the potential oncogenic function in LUAD. Meanwhile, qRT-PCR, CCK8, wound healing, and transwell assays were employed to determine the potential biological function of AC087588.2 in LUAD progression.

## Materials and Methods

### TCGA Datasets

Transcription and clinical information of LUAD was downloaded from TCGA (https://portal.gdc.com) (Tomczak et al., 2015). RNA-seq gene expression data of workflow type FPKM were transformed into TPM format and log2 transformation for further study. The timeROC analysis was used to compare the predictive accuracy of the AC087588.2 gene in LAUD. Clinical information of the LUAD patients consisted of the pathological stage, TNM stage, smoker, OS, DSS, and PFS.

### Cox Regression Analysis and Kaplan–Meier Survival Analysis

We utilized Cox regression analysis to examine the correlation between AC087588.2 expression and overall survival and disease-specific survival of patients using the TCGA databases. The Kaplan–Meier method was used to assess the difference between high- and low-risk groups based on the best separation of AC087588.2 expression employing R packages of survminer and survival.

### Gene Set Enrichment Analysis

In the present research, we utilized the LinkeDomics database (http://www.linkedomics.org/login.php) to obtain the co-expression genes of AC087588.2 in LUAD. KEGG and GO were employed to assess the potential functions of AC087588.2 in LUAD with the R package ClusterProfiler (Yu et al., 2012). GSEA software was used to explore the potential signaling pathway involved by AC087588.2 in LUAD (Subramanian et al., 2005; Yu et al., 2012; Vasaikar et al., 2018).

### The Target Gene of miR-30a-5p Predicted by Various Databases

StarBase (https://starbase.sysu.edu.cn/) is a database that includes the miRNA-ceRNA, miRNA-ncRNA, and protein–RNA interaction networks from large-scale CLIP-Seq data. In this manuscript, we used StarBase to predict the target gene of miR-30a-5p. StarBase was also utilized to determine the relationship between miR-30a-5p expression and AC087588.2 in LUAD (Cho et al., 2013; Dweep et al., 2014; Li et al., 2014; Chen and Wang, 2020).

### Immune Infiltration Analysis by ssGSEA

In this study, the ssGSEA method was employed to analyze the association between AC087588.2 expression and the infiltration of 24 tumor-infiltrating lymphocytes (TILs) in LUAD (Bindea et al., 2013)

### Cell Culture Conditions

Lung cancer cell lines, including one human normal bronchial epithelial cell (BEAS-2B) and three human lung adenocarcinoma cells (H1975, SPC-A1, H1299, and A549 cells), were purchased from the Chinese Academy of Sciences Cell Bank (CASCB, China) and cultured in RPMI 1640 medium (Corning) including 10% fetal bovine serum (FBS) and 1% penicillin/streptomycin at 37°C an atmosphere containing 95% air and 5% CO_2_.

### siRNA and Transfection

SiRNA for AC087588.2 and the matched negative controls were designed and synthesized by RiboBio (Guangzhou, China). Lipofectamine™ 3000 Reagent (Invitrogen, United States) was used to transfect siRNA according to the manufacturer’s instructions. The primer used in this study is as follows: AC087588.2 siRNA#1: GCC​TTG​GTC​ATG​AAA​CGA​TTA.

### Quantitative Real-Time PCR

The qRT-PCR assay was performed as documented (Pan et al., 2020). The primer sequences are as follows: AC087588.2 -F: GCA​CTT​ACT​TTA​TAG​CAG​CAA, AC087588.2 -R: ATA​AAT​ATG​GTT​TCT​CAA​GT; β-actin-F: CTTCGCGGGCGACGAT, β-actin-R: CCA​TAG​GAA​TCC​TTC​TGA​CC. The expression quantification was obtained with the 2−ΔΔCt method.

#### Cell Migration Assay

For the transwell migration assay, 2.5×10^4^ cells/well in 100 μL serum-free medium were plated in a 24-well plate chamber insert, and the lower chamber was filled with 10% FBS. After incubation for 24 h, cells were fixed with 4% paraformaldehyde, washed, and then stained with 0.5% crystal violet for further pictures captured.

### CCK8 Assay

We seeded cells in 96-well plates at 2.5 × 10^3^ per well in 100 μl of complete medium and 10 μl of CCK-8 reagent (RiboBio, Guangzhou, China) for 1 h each day after 3 days of culture. We then used a microplate to measure the absorbance of each well at 450 nm. Each sample was evaluated in triplicate.

### Statistical Analyses

The significance of the data between two experimental groups was determined by Student’s *t*-test, and multiple group comparisons were analyzed by one-way ANOVA. *p* < 0.05 (*), *p* < 0.01 (**), and *p* < 0.001 (***) were significant.

## Results

### Differential Expression of AC087588.2 in LUAD Patients

**FIGURE 1:**
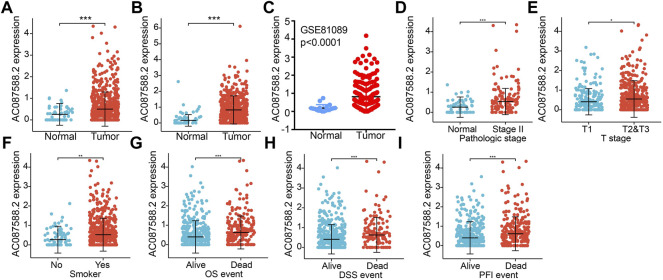
AC087588.2 was overexpressed in LUAD. **(A–C)** AC087588.2 was overexpressed in LUAD and LUSC examined by TCGA and GEO datasets. **(D–I)** Correlation between AC087588.2 expression and clinical parameters, including the pathological stage, T stage, smoking, overall survival event, disease-specific survival event, and progression-free survival event. *p < 0.05, **p < 0.01, and ***p < 0.001.

To examine AC087588.2 expression in LUAD, we analyzed AC087588.2 expression data in TCGA-LUAD and uncovered that AC087588.2 was upregulated in 535 tumor tissues compared to 59 normal lung tissues in LUAD and upregulated in 502 tumor tissues compared to 49 normal lung tissues in LUSC (Figures 1A,B). Further analyses of one dataset of GEO obtained similar results (Figure 1C). To determine the potential function of AC087588.2 in the development of LUAD, we analyzed the correlation between AC087588.2 expression and various clinical features. The analysis indicated that AC087588.2 was significantly associated with advanced pathological stage, T stage, smoking, OS event, DSS event, and PFS event (Figures 1D–I).

### AC087588.2 Modulates Cell Proliferation and Migration of LUAD Cells

**FIGURE 2:**
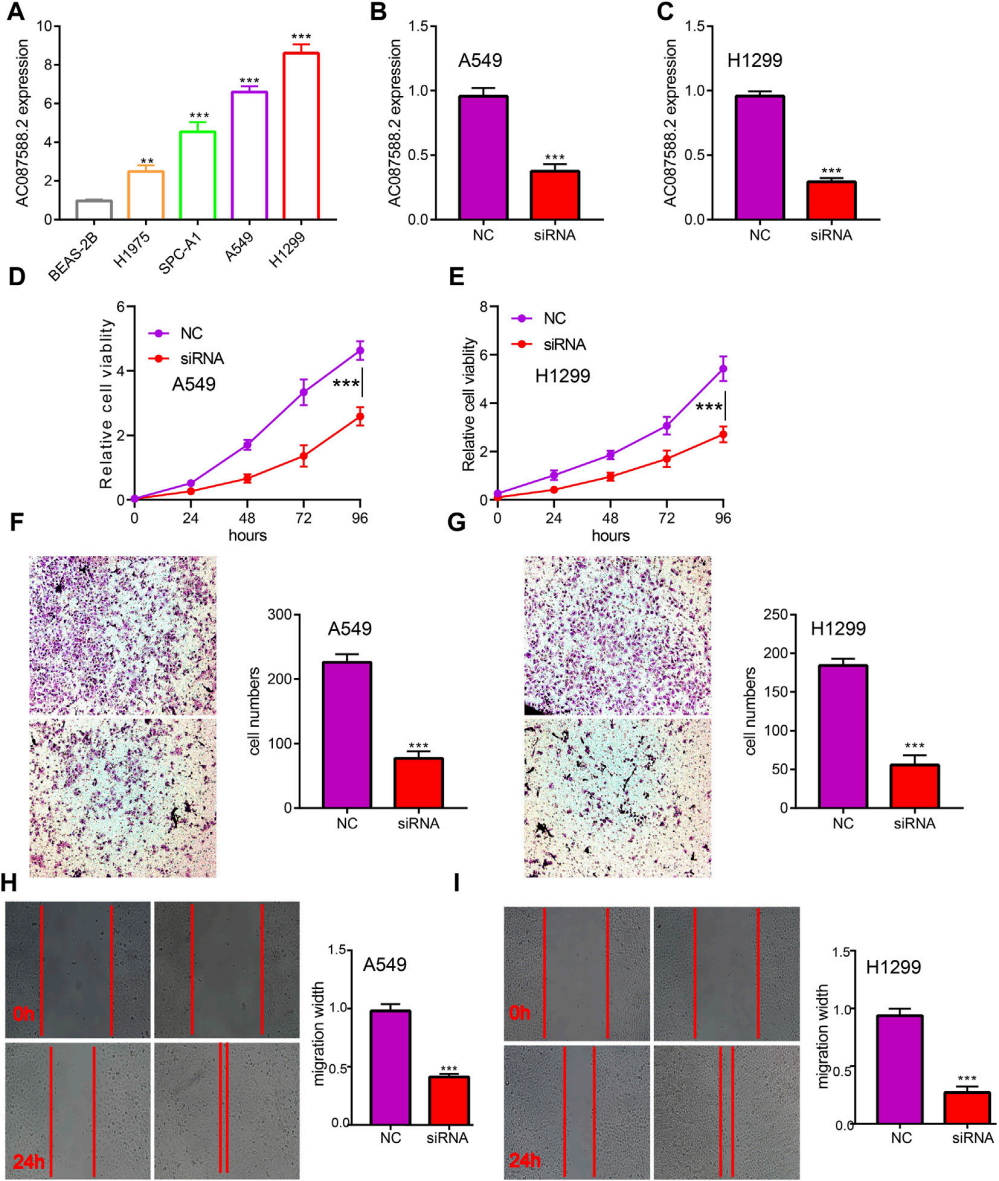
AC087588.2 modulates LUAD cell proliferation and migration *in vitro*. **(A)** The expression of AC087588.2 in LUAD cell lines examined by qPCR. **(B–C)** qPCR assay examined the knockdown efficiency of AC087588.2 in A549 and H1299 cells **(D–I)** AC087588.2 knockdown inhibited cell proliferation and migration examined by CCK8, transwell and wound healing assay. **p < 0.01, and ***p < 0.001. NC = negative control, siRNA = AC087588.2 siRNA.

To explore the biological role of AC087588.2 in LUAD, we found that AC087588.2 expression was significantly upregulated in H1975, SPC-A1, H1299, and A549 cell lines (Figure 2A). Moreover, specific siRNA for AC087588.2 was used to knock down the AC087588.2 expression (Figures 2B,C). It was shown that the depletion of AC087588.2 inhibited the proliferative capacity of LUAD cells (Figures 2D,E). Moreover, transwell and wound healing assay confirmed that AC087588.2 knockdown inhibited the cell migration of LUAD (Figures 2F–I). These data indicated that AC087588.2 is functionally important in regulating LUAD progression.

### Association Between AC087588.2 Expression and Clinical Outcome in LUAD

**FIGURE 3:**
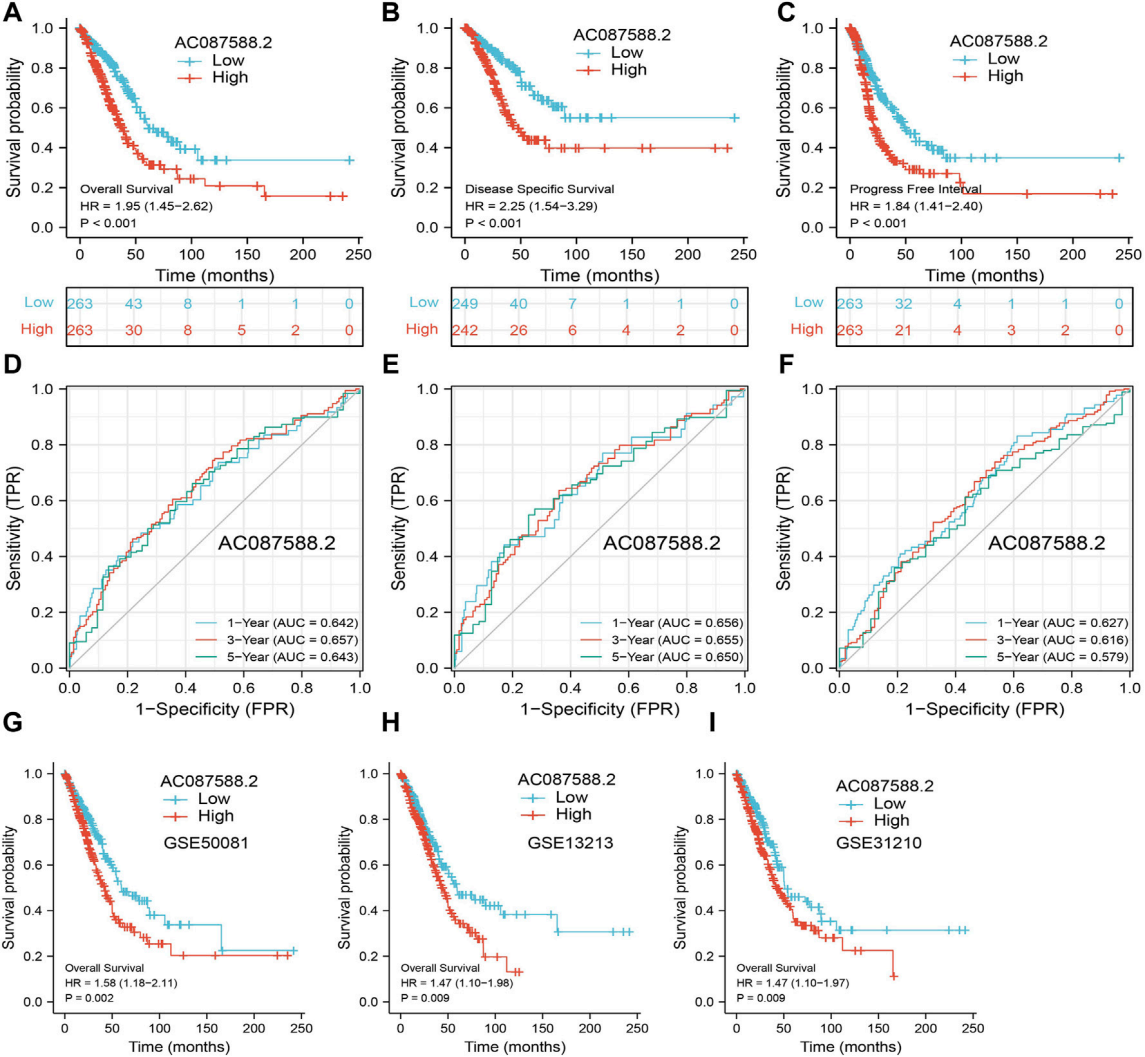
ROC and Kaplan–Meier curves of AC087588.2. **(A–C)** OS, DSS, and PFS of AC087588.2 in LUAD determined using the TCGA-LUAD dataset. **(D–F)** ROC curve of AC087588.2 in LUAD determined using the TCGA-LUAD dataset. **(G–I)** Validation of overall survival of AC087588.2 in lung cancer by GEO datasets.

**FIGURE 4:**
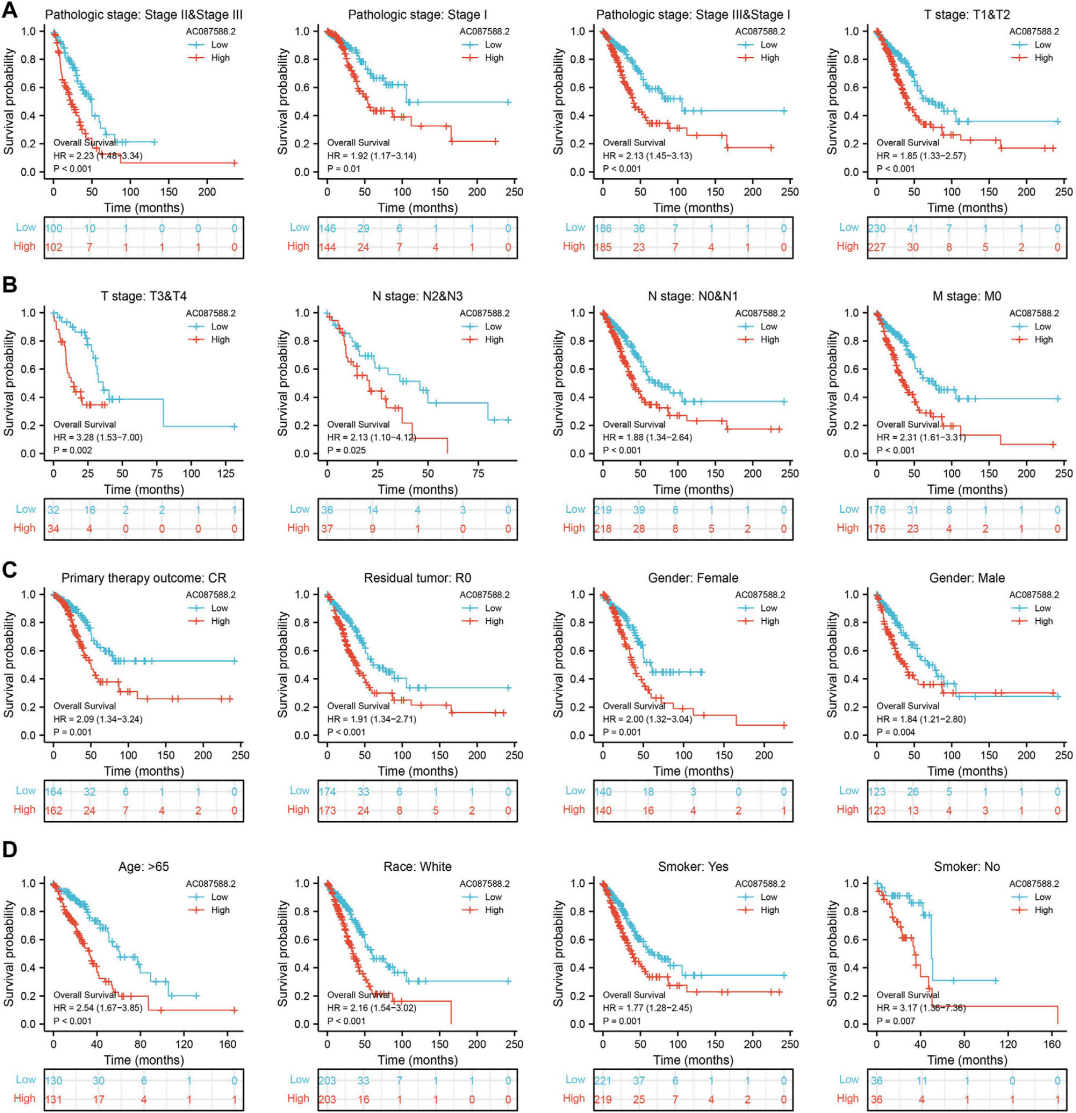
Overall survival of AC087588.2 based on the diverse subgroup. **(A–D)** Overall survival of AC087588.2 based on the diverse subgroup, including stages II and III, stage I, stages I and III, T1 and T2, T3 and T4, N0 and N1, N2 and N3, M0, primary: CR, R0, female, male, age >65, smoker.

Next, to determine the correlation between AC087588.2 expression and clinical outcome of LUAD patients, the TCGA-LUAD datasets were employed. Based on the median expression of AC087588.2 in LUAD, the expression level of AC087588.2 in LUAD patients was divided into two groups with high and low expression. Results suggested that upregulation of AC087588.2 was significantly associated with poor OS, DSS, and PFS in patients with LUAD (Figures 3A–C). Based on the time-dependent ROC, the AC087588.2 expression level had a relatively good performance in predicting the 1-, 3-, and 5-year OS, DSS, and PFS in LUAD patients (Figures 3D–F). We also utilized GEO datasets validation the prognosis of AC087588.2 in lung cancer and obtained the same results (Figures 3G–I). Moreover, an overall survival analysis was conducted to determine the prognostic value of AC087588.2 in different subgroups of LUAD patients stratified by stages II and III, stage I, stages I and III, T1 and T2, T3 and T4, N0 and N1, N2 and N3, M0, primary: CR, R0, female, male, age >65, and smoker. Results suggested that increased AC087588.2 level is associated with poor prognosis in patients with LUAD (Figures 4A–D).

### AC087588.2 as an Independent Risk Factor

**FIGURE 5:**
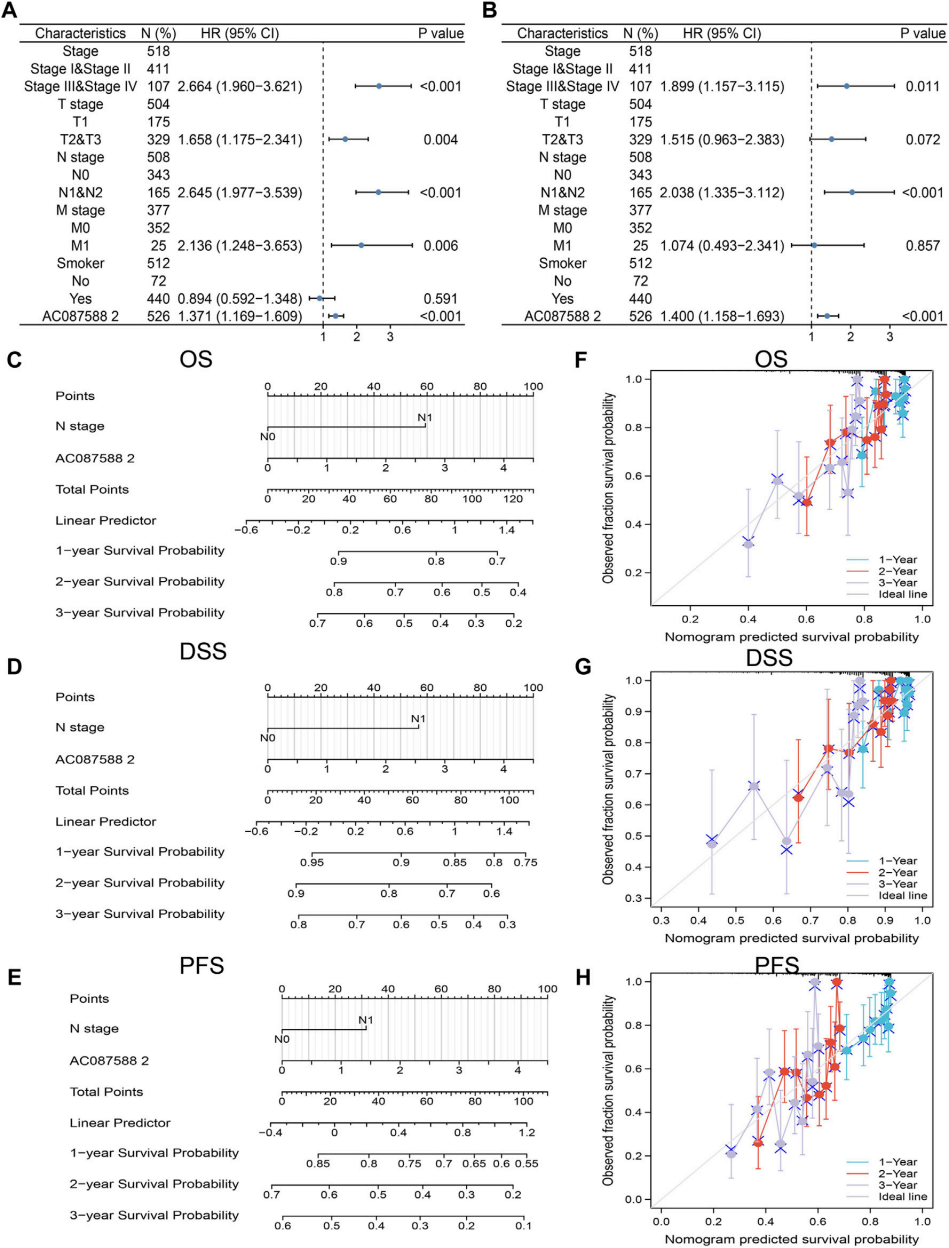
Univariate and multivariate Cox regression analysis. **(A,B)** Univariate and multivariate Cox regression analysis in LUAD. **(C–E)** The nomogram was developed by integrating the AC087588.2 expression and T stage in the TCGA databases. **(F–H)** Predicting abilities of the nomogram.

To determine whether AC087588.2 is an independent factor for the prognosis of LUAD, we conducted the univariate and multivariate Cox regression analyses based on the TCGA-LUAD. Results suggested that the AC087588.2 expression, pathological stage, and N stage could be independent risk factors for LUAD (Figures 5A,B). Then, we constructed the nomograms using the above independent prognosis factors (N stage and AC087588.2 expression) to predict 1-, 3-, and 5-year OS, DSS, and PFS of each LUAD patient (Figures 5C–E). The calibration plot of survival suggested that this nomogram could predicate OS, DSS, and PFS (Figures 5F–H).

### KEGG Enrichment Analysis for AC087588.2 in LUAD

**FIGURE 6:**
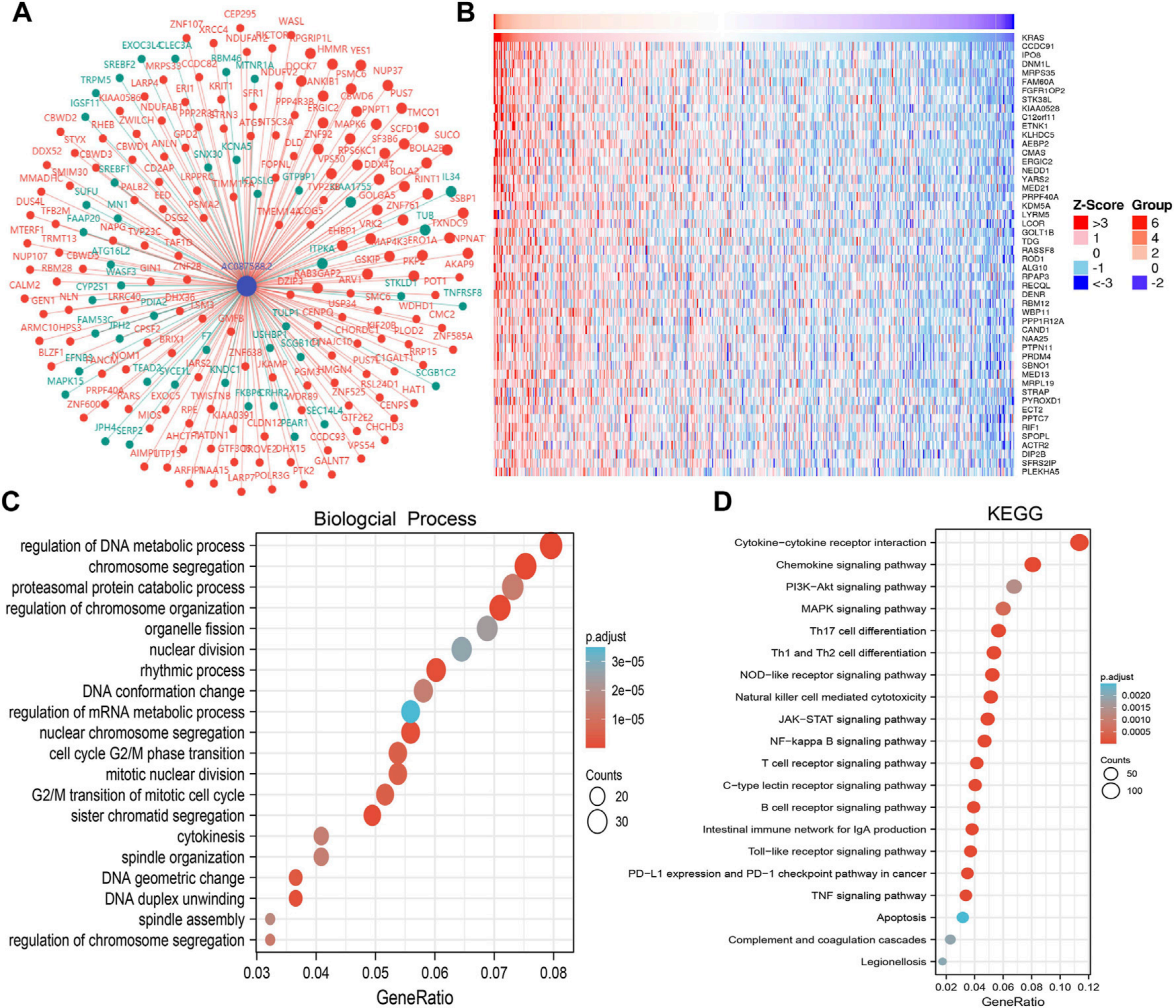
KEGG enrichment analysis of AC087588.2. **(A,B)** Gene–gene interaction network of AC087588.2 in LUAD. **(C,D)** GO and KEGG enrichment analysis of AC087588.2 in LUAD.

**FIGURE 7:**
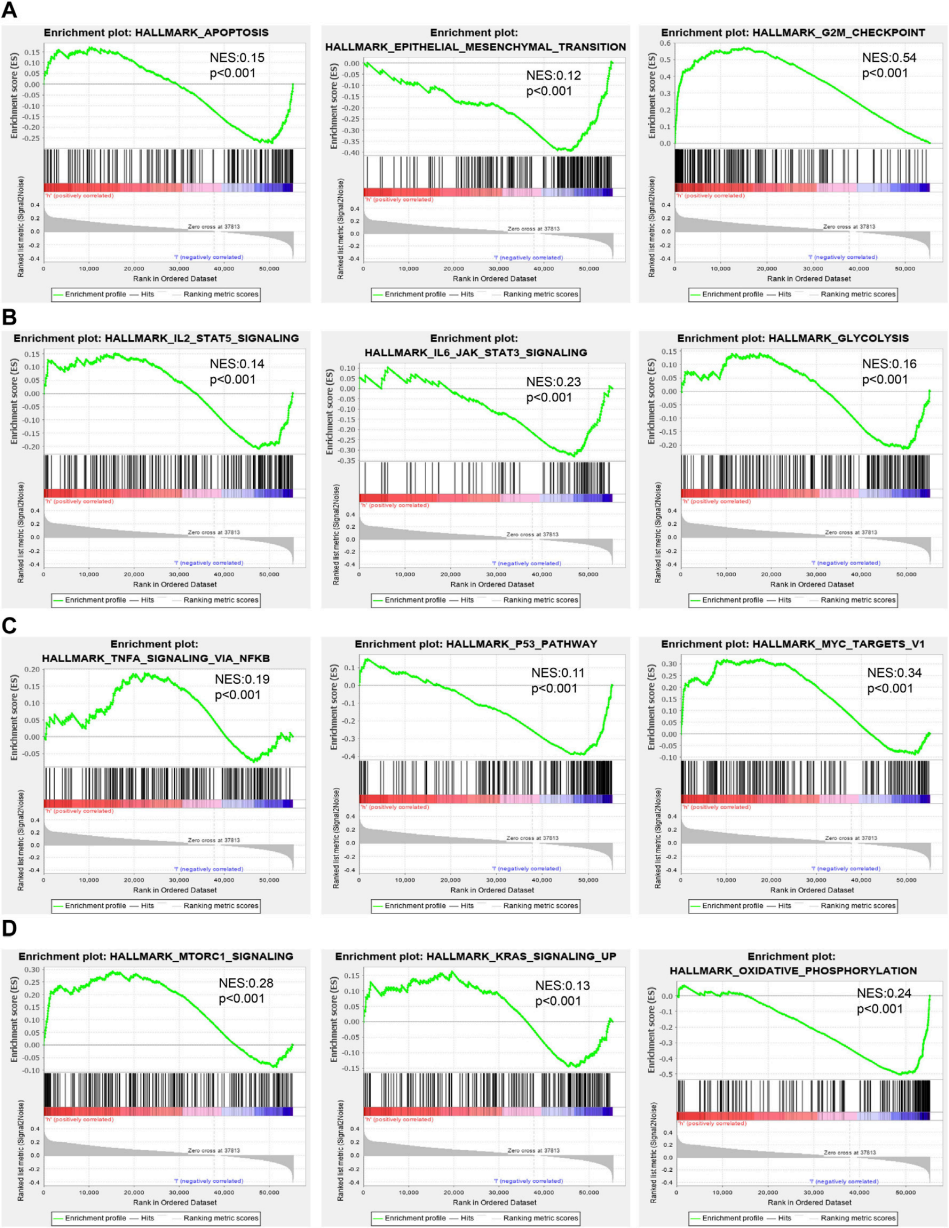
GSEA identification of AC087588.2-related signaling pathways. **(A–D)** Identification of AC087588.2-related signaling pathways by GSEA software.

To examine the potential biological functions of AC087588.2 in LUAD, we performed KEGG enrichment analysis on genes that were significantly positively correlated with AC087588.2 expression based on the TCGA-LAUD dataset (Figures 6A,B). The GO functional analyses suggested that AC087588.2 was mainly involved in the regulation of DNA metabolic process, chromosome segregation, and proteasomal protein catabolic process regulation of chromosome organization (Figure 6C). Meanwhile, the KEGG pathway analyses confirmed that AC087588.2-related pathways involve the PI3K−Akt signaling pathway, MAPK signaling pathway, JAK−STAT signaling pathway, and TNF signaling pathway (Figure 6D). Gene set enrichment analysis (GSEA) also showed that AC087588.2 was mainly involved in the cell apoptosis, EMT, G2/M checkpoint, glycolysis, p53, MYC targets, and oxidative phosphorylation (Figures 7A–D).

### Correlation Between AC087588.2 Expression and Immune Infiltration

**FIGURE 8:**
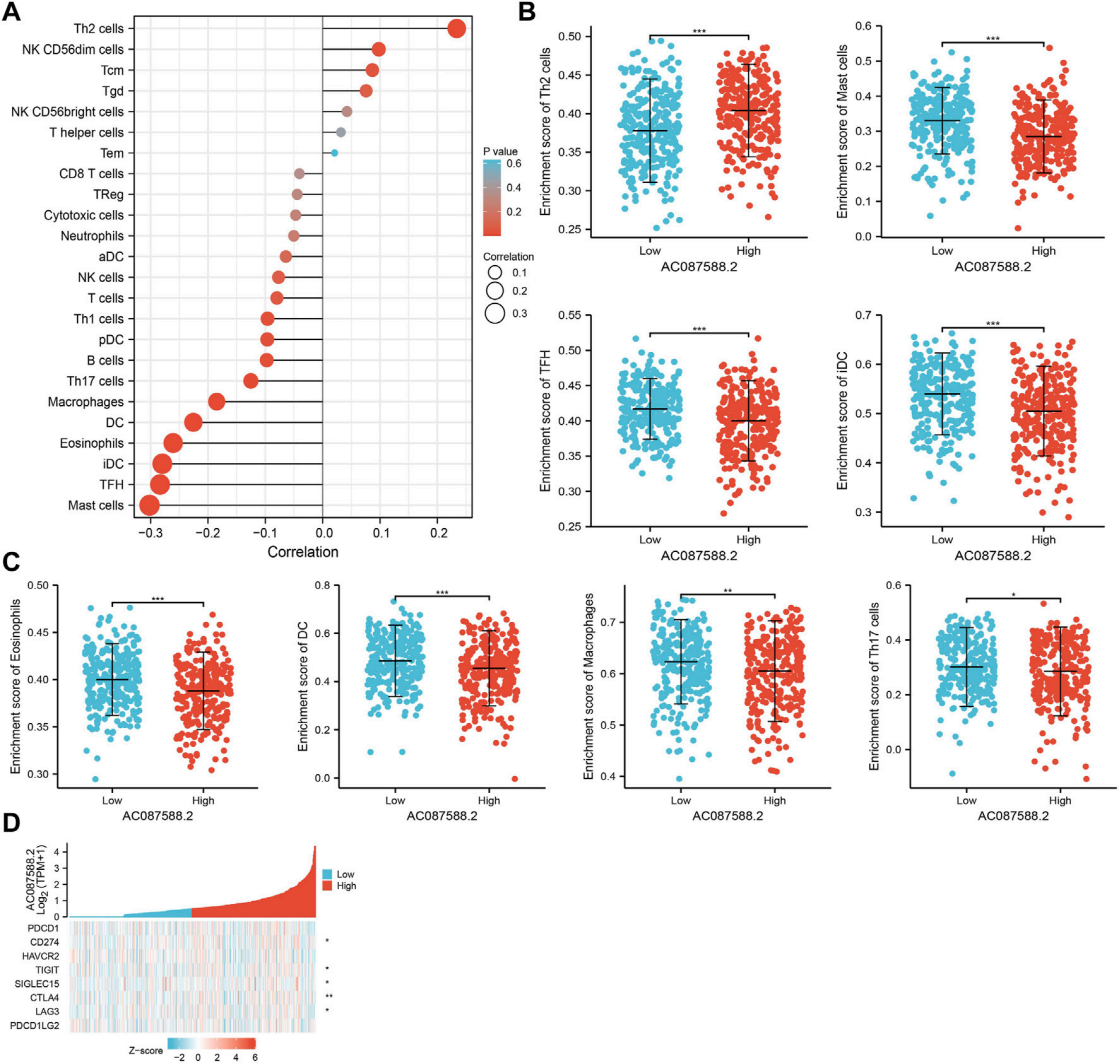
Association between AC087588.2 expression and immune cell infiltration in LUAD. **(A–C)** Correlation between AC087588.2 expression and immune cell infiltration in LUAD. **(D)** Correlation between AC087588.2 expression and immune checkpoint-related genes in LUAD. *p < 0.05, **p < 0.01, and ***p < 0.001.

Immune infiltration has a crucial role in LUAD development [25]. Therefore, we explore the correlation between AC087588.2 expression and the infiltration levels of 24 types of immune cells in LUAD using the ssGSEA method. Results suggested that the AC087588.2 expression in LUAD was positively related to the infiltration of Th2 cells and negatively associated with the abundance of B cells, Th17 cells, macrophages, DC, eosinophils, iDC, TFH, and mast cells in LUAD (Figures 8A–C).

Given that immune checkpoints play a crucial role in tumor immunosuppression, we analyzed the correlation between AC087588.2 expression and that of the immune checkpoint-related genes of *CD274*, *CTLA4*, *HAVCR2*, *LAG3*, *PDCD1*, *PDCD1LG2*, *TIGIT*, and *SIGLEC15* in LUAD using Pearson’s correlation analysis. AC087588.2 expression was significantly positively correlated with the expression of *CD274*, *CTLA4*, *LAG3*, *TIGIT*, and *SIGLEC15* in this analysis (Figure 8D). These results confirmed that AC087588.2 played a crucial role in immune infiltration in LUAD.

### AC087588.2-Related miRNA–mRNA Network in LUAD

**FIGURE 9:**
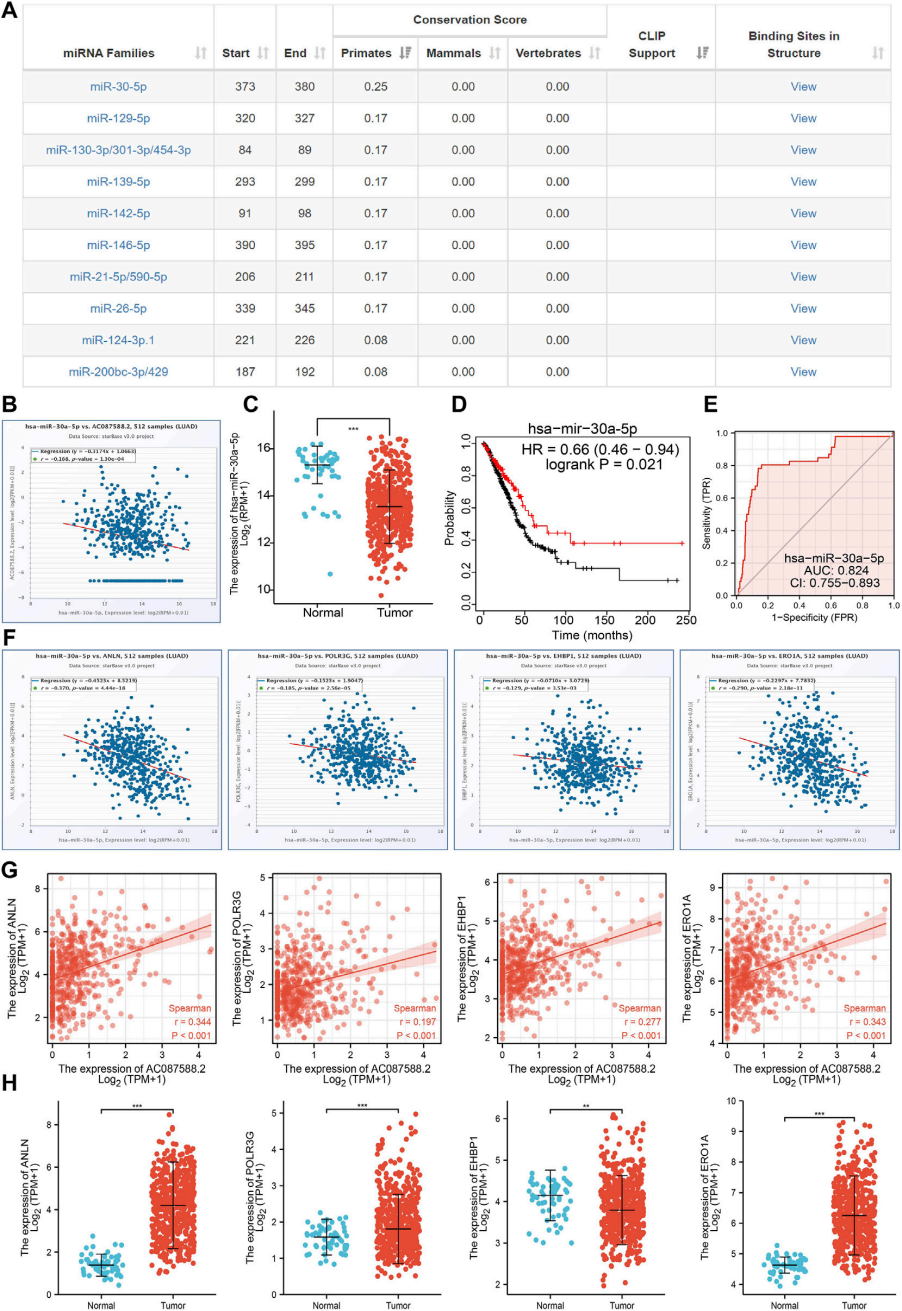
Analysis of the potential miRNAs of AC087588.2. **(A)** Potential miRNAs of AC087588.2 determined by the Anbolnc2 database. **(B)**. Correlations between AC087588.2 expression and miR-30a-5p in LUAD. **(C)** Expression level of miR-30a-5p in LUAD. **(D)** Overall survival of miR-30a-5p in LUAD. **(E)** ROC curve of miR-30a-5p in LUAD. **(F)** Correlations between the miR-30a-5p expression and ANLN, POLR3G, EHBP1, and ERO1A in LUAD. **(G)** Correlations between the AC087588.2 expression and ANLN, POLR3G, EHBP1, and ERO1A in LUAD. **(H)** RNA level of ANLN, POLR3G, EHBP1, and ERO1A in LUAD.

To further explore the AC087588.2-mediated downstream regulatory mechanism involved in LUAD progression, we used the Annolnc2 (http://annolnc.gao-lab.org/) database to predict the potential miRNAs binding with AC087588.2. We obtained 10 miRNAs (Figure 9A) (Ke et al., 2020). Based on the competitive endogenous RNAs theory, lncRNA should be positively correlated with mRNA and negatively correlated with miRNA. Among all the 10 miRNAs, only miRNA-30a-5p negatively correlated with AC087588.2 in LUAD (Figure 9B). Moreover, we found that has-miR-30a-5p had low expression in LUAD, which correlated with poor prognosis in patients with LUAD (Figures 9C,D). ROC curve analysis showed that the AUC value of has-miR-30a-5p is 0.824 (Figure 9E). Therefore, we selected has-miR-30a-5p to conduct downstream analysis.

### Identification of the Potential Downstream Target of AC087588.2/miR-30a-5p in LUAD

**FIGURE 10:**
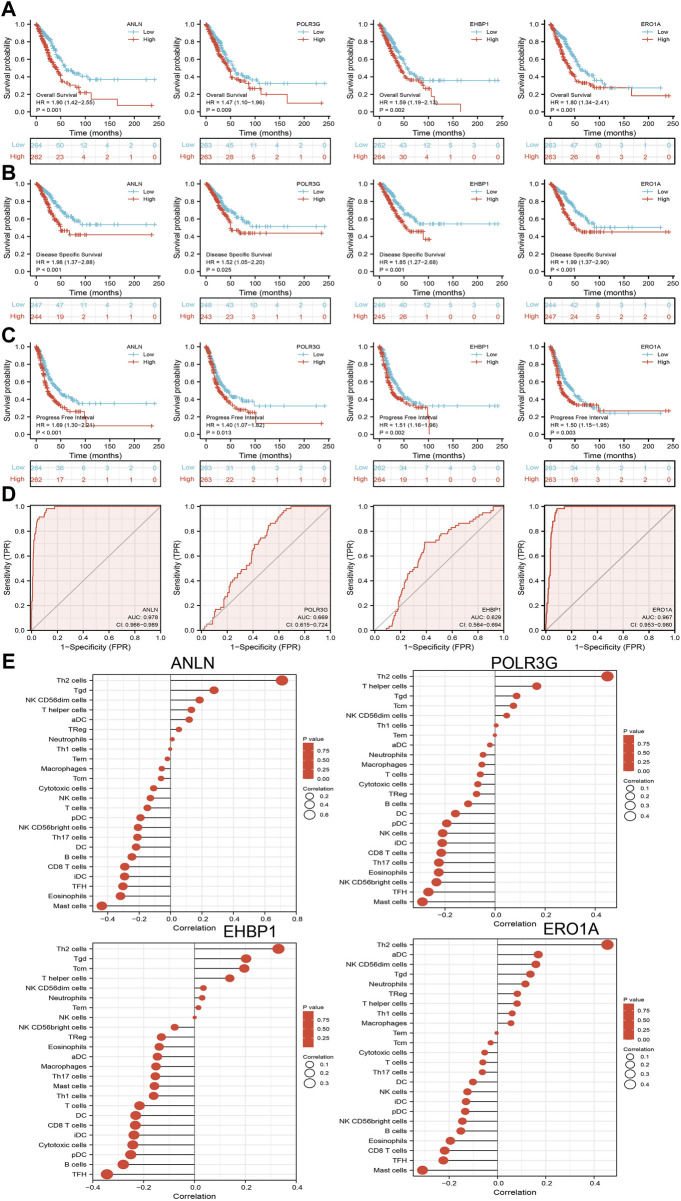
Analysis of the prognostic and diagnostic value of ANLN, POLR3G, EHBP1, and ERO1A in LUAD. **(A–C)** OS, DSS, and PFS of ANLN, POLR3G, EHBP1, and ERO1A in LUAD. **(D)** ROC curve of ANLN, POLR3G, EHBP1, and ERO1A in LUAD. **(E)** Correlation between ANLN, POLR3G, EHBP1, and ERO1A expression and immune infiltration levels of 24 immune cells in LUAD.

We further investigated the target genes of miR-30a-5p that play critical roles in the progression of LUAD. First, we predicted the target in StarBase, miRDB, miRWalk, and miRGator (Cho et al., 2013; Dweep et al., 2014; Li et al., 2014; Chen and Wang, 2020). According to the prediction of target genes, we found that only four genes (ANLN, POLR3G, EHBP1, and ERO1A) were negatively correlated with the miR-30a-5p expression in LUAD (Figure 9F). Importantly, the expression levels of ANLN, POLR3G, EHBP1, and ERO1A were positively correlated with those of AC087588.2in LUAD (Figure 9G). Furthermore, we employed the TCGA to explore the expression level and prognosis in LUAD. We found that the expression levels of ANLN, POLR3G, EHBP1, and ERO1A were significantly increased in LUAD and associated with OS, DSS, and PFS in patients with LUAD (Figure 9H; 10A–C). ROC curve was utilized to examine the diagnostic value of ANLN, POLR3G, EHBP1, and ERO1A in LUAD, the AUC (area under the curve) of which were 0.978, 0.669, 0.629, and 0.927, respectively (Figure 10D), suggesting that ANLN, POLR3G, EHBP1, and ERO1A were a potential prognostic and diagnostic biomarker in LUAD. Finally, we used the ssGSEA method to determine the correlations between ANLN, POLR3G, EHBP1, and ERO1A and 24 types of tumor-infiltrating immune cells. Results confirmed that the expression levels of ANLN, POLR3G, EHBP1, and ERO1A were positively correlated with the cell infiltrating of Th2 cells in LUAD (Figure 10E).

## Discussion

At present, common treatments for lung cancer mainly include surgical resection, radiation-chemotherapy, and immunotherapy, but the therapeutic effect is not ideal. Consequently, it is urgent to identify novel cancer biomarkers and understand the potential molecular mechanisms involved in LUAD initiation and progression. It has been confirmed that lncRNAs play an important role in modulating cell proliferation, cell apoptosis, and cancer progression (Dong et al., 2018).

Increasing evidence demonstrated the functional and clinical role of lncRNAs involved in the progression (Jia et al., 2019; Xue et al., 2021). For example, it has been shown that the increased AC079630.4 expression is related to the progression and prognosis in lung cancer (Wang et al., 2021). Previous studies indicated that LncRNAs have clinical predictor value in several tumors (Chao and Zhou, 2019). For instance, Wang et al. found that linc8087 was downregulated in NSCLC and its lower expression was related to adverse prognosis in patients with NSCLC (Qi et al., 2021).

In the current study, we uncovered that AC087588.2 was overexpressed in LUAD, and its higher expression was correlated with adverse OS, DSS, and PFS in patients with LUAD. Additionally, ROC curve analysis confirmed that the AUC value of AC087588.2 is 0.888. Results suggested that AC087588.2 could serve as a sensitive indicator to predict the prognosis of the patients, indicating the value of AC087588.2 as a prognostic biomarker for LUAD. Multivariate analysis indicated that the AC087588.2 expression was an independent prognostic indicator for the OS, DSS, and PFS of LUAD patients.

Previous studies reported that lncRNA plays an important role in the EMT and cell cycle (Jia et al., 2019; Geng et al., 2021). For example, it has been confirmed that lncRNA-JPX modulates cell proliferation and migration by sponging miR-33a-5p to increase Twist1 expression (Pan et al., 2020). In this study, we found that AC087588.2 was mainly involved in the cell apoptosis, EMT, G2/M checkpoint, glycolysis, p53, MYC targets, and oxidative phosphorylation.

It has been confirmed that lncRNA plays a central role in facilitating tumor progression and immune escape (Zhang et al., 2020). For example, lncRNA GATA3-AS1 promoted BRCA immune escape by stabilizing PD-L1 (Zhang et al., 2020). In this finding, we demonstrated that the AC087588.2 expression in LUAD was positively associated with the infiltration of Th2 cells and negatively correlated with the abundance of B cells, Th17 cells, macrophages, DC, eosinophils, iDC, TFH, and mast cells in LUAD. Given that immune checkpoints play a crucial role in tumor immunosuppression, we analyzed the correlation between the AC087588.2 expression and that of the immune checkpoint-related genes of *CD274*, *CTLA4*, *HAVCR2*, *LAG3*, *PDCD1*, *PDCD1LG2*, *TIGIT*, and *SIGLEC15* in LUAD using Pearson’s correlation analysis. The AC087588.2 expression was significantly positively correlated with the expression of *CD274*, *CTLA4*, *LAG3*, *TIGIT*, and *SIGLEC15* in this analysis (Figure 8D). These results confirmed that AC087588.2 played a crucial role in immune infiltration in LUAD.

Finally, we uncovered that AC087588.2 was significantly upregulated in NSCLC cell lines, and the depletion of AC087588.2 markedly inhibited cell proliferation and migration in LUAD.

We also utilized various databases to identify the potential target gene of AC087588.2/miRNA-30a-5p in LUAD, including the *ANLN*, *POLR3G*, *EHBP1*, and *ERO1A*. Subsequent expression and prognosis analysis confirmed that ANLN, POLR3G, EHBP1, and ERO1A were significantly greater in LUAD tissues than those in the normal LUAD tissues, and the upregulation of ANLN, POLR3G, EHBP1, and ERO1A expression was associated with poor prognosis in patients with LUAD. In conclusion, this finding provided a possible comprehensive view of the AC087588.2-mediated ceRNA network in LUAD, thereby highlighting its potential role in diagnosis and therapy. Finally, we uncovered that AC087588.2 was significantly upregulated in NSCLC cell lines and depletion of AC087588.2 markedly inhibited cell proliferation and migration in LUAD.

This study improves our understanding of the correlation between AC087588.2 and LUAD, but some limitations still exist. First, although we explored the correlation between AC087588.2 and immune infiltration in LUAD patients, there is a lack of experiments that validate the function of AC087588.2 in the tumor microenvironment regulation of LUAD. Second, we confirmed that the knockdown of AC087588.2 inhibited cell proliferation and cell migration of LUAD. However, the potential molecular mechanisms of AC087588.2 in cancer progression need to be explored in further studies.

## Conclusion

Our data confirmed that AC087588.2 could serve as a promising diagnostic and prognostic biomarker for LUAD patients.

## Data Availability

The original contributions presented in the study are included in the article/Supplementary Material; further inquiries can be directed to the corresponding authors.
